# Drug Delivery Systems Based on Dendritic-Cell-Derived Exosomes

**DOI:** 10.3390/pharmaceutics17030326

**Published:** 2025-03-03

**Authors:** Lihua Chen, Jie Zhang, Yueyan Huang, Xiaoqin Zhang, Guoqing Zhang, Shuaizhi Kong, Jianqing Gao, Xiaojuan Zhang, Baoyue Ding

**Affiliations:** 1 College of Pharmaceutical Science, Zhejiang University of Technology, Hangzhou 310014, China; chenlihua0226@163.com (L.C.); zgq2119@163.com (G.Z.); 221122070233@zjut.edu.cn (S.K.); 2 Jiaxing Key Laboratory for Photonanomedicine and Experimental Therapeutics, Department of Pharmaceutics, College of Medicine, Jiaxing University, No. 118 Jiahang Road, Jiaxing 314001, China; zhangjiepharm@zjxu.edu.cn (J.Z.); hyylinda@163.com (Y.H.); qin@zjxu.edu.cn (X.Z.); gaojianqing@zju.edu.cn (J.G.); 3 Zhejiang Province Key Laboratory of Anti-Cancer Drug Research, Institute of Pharmaceutics, College of Pharmaceutical Sciences, Zhejiang University, Hangzhou 310058, China

**Keywords:** dendritic cells, exosomes, drug delivery system, immunotherapy

## Abstract

Exosomes, spherical lipid-bilayered particles secreted by cells, have recently emerged as a novel and highly promising drug delivery system, attracting extensive attention in the field of biomedical research. Dendritic-cell-derived exosomes (DC-Exos) possess surface protein and ligands characteristic of DC cells, such as functional MHC-I and MHC-II, CD80, CD86. These components play a crucial role in immune responses, facilitating antigen uptake, presentation, and the activation of antigen-specific CD4 and CD8 T cells. These properties make them striking and excellent drug delivery vehicles for use in various immune diseases and cancer therapy. This review summarizes and discusses the characteristics, current methods and types of drug loading of DC-Exos. Its surface modifications and application in disease treatment were also discussed, aiming to motivate the development of exosome-based theranostic nanoplatforms and nanotechnology for improved healthcare treatments.

## 1. Introduction

Drug delivery systems (DDS) are methods designed to selectively deliver drugs to specific organs, tissues, even cells, for therapeutic purposes. The primary objective is to enhance the pharmacological activity of drugs while addressing challenges such as low solubility, drug aggregation, bioavailability, and biodistribution. Additionally, DDS aim to improve selectivity and minimize the toxic and side effects of drugs [1]. Current DDS have made significant advancements over traditional DDS by addressing several limitations. These improvements include enhanced drug selectivity, controlled release mechanisms, increased bioavailability, reduced side effects, and better adherence, particularly in patients with chronic inflammation who often experience premature metabolic excretion [2]. However, current DDS still face several challenges that need to be addressed, including lower biocompatibility, potential toxicity, the exogenous origin of synthetic materials, immunogenicity, and a short blood circulation time [3]. Drug carriers can be modified with hydrophilic polymers, such as polyethylene glycol (PEG), to reduce phagocytosis by the reticuloendothelial system (RES) and enhance their circulation time in vivo [4]. However, due to the high hydrophilicity of PEG, drug carriers modified with PEG often exhibit low cell adhesion. This limitation hinders the interaction of drug carriers with cell membranes and reduces cellular internalization. Furthermore, exogenous DDS possess a degree of immunogenicity, which can trigger an immune response in the body [5]. Therefore, the search for more efficient DDS remains essential to minimize immune clearance by the body and enhance drug delivery efficiency.

Extracellular vesicles (EVs) are micro- and nano-scale vesicles secreted by cells, which can be classified as exosomes, microvesicles, and apoptotic bodies, based on their size, properties, and biogenesis [6,7]. In recent years, exosomes characterized by a bilayer lipid membrane structure have emerged as a focal point of research in the field of drug delivery. This is largely due to their excellent biocompatibility, minimal immunogenicity, low toxicity, and other beneficial properties. Compared to synthetic delivery carriers, exosomes offer distinct advantages as DDS. Firstly, exosomes exhibit excellent biocompatibility because they naturally occur in the body. Almost all cells can secrete exosomes, and serum proteins bind weakly to them. This characteristic enhances their biocompatibility compared to other DDS, allowing for an extended circulating half-life of drugs [8]. Additionally, exosomes can deliver therapeutic agents directly into the cytoplasm by bypassing the endosomal pathway, thereby avoiding lysosomal degradation [9]. Secondly, exosomes are endogenous in origin, which renders them less immunogenic and enables them to easily cross biological barriers, including the blood–brain barrier [10]. Thirdly, exosomes are abundantly present in various body fluids, including blood, cerebrospinal fluid, urine, saliva, and breast milk. This widespread occurrence suggests that exosomes are well tolerated by the body. Additionally, exosomes as DDS protect therapeutic agents from degradation and extend their circulating half-life, thereby enhancing the efficacy of the drugs [11]. Exosomes are released by a diverse array of cell types, including macrophages, mesenchymal stem cells, tumor cells, lymphocytes, and dendritic cells (DCs) [12]. Notably, DC-derived exosomes (DC-Exos) exhibit a molecular composition that closely resembles that of DCs. Furthermore, DC-Exos encapsulate genetic materials, soluble proteins, and lipids derived from DCs. Consequently, DC-Exos not only functions as a vehicle for drug delivery but also plays a pivotal role in mediating various immune responses, akin to the functions of DCs [13].

In this review, we establish a comprehensive overview of the current drug loading types and methods of DC-Exos, highlights the surface modifications and the application of DC-Exos in disease treatment, and looks at the application challenges and future trends of DC-Exos in DDSs, aiming to motivate the development of exosome-based theranostic nanoplatforms and nanotechnology for improving healthcare treatments.

## 2. Components and Functions of DC-Exos

**Figure 1:**
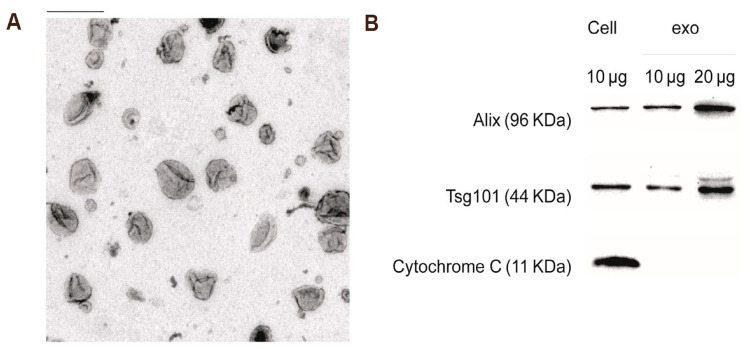
Characterization of DC-Exos. (**A**) Transmission electron microscopic image of DC-Exos (scale bar  =  200 nm). (**B**) Western blot analysis for detecting the expression of exosome biomarkers and cellular protein in DC-Exos. Total protein was loaded for DC cell lysates (10 μg) and DC-Exos (10 μg and 20 μg) [27].

DCs, which are derived from bone marrow progenitor cells [14], were first identified by Ralph Steinman and Zanvil Cohn in 1973 [15]. They are recognized as the most potent antigen-presenting cells in humans and other mammals. The primary distinction between DC-Exos and exosomes derived from other sources lies in their composition and function. DC-Exos not only possess the molecular characteristics typical of general exosomes but also carry a variety of immunocompetent molecules. Furthermore, unlike exosomes from other origins, DC-Exos can actively participate in and regulate numerous immune-related responses, thereby influencing both the intensity and direction of the immune response [16]. For instance, like DCs, DC-Exos are abundant in proteins, lipids, and genetic materials [17]. The primary proteins found in DC-Exo include intercellular adhesion molecule 1 (ICAM-1), integrin α and β-chains (αMβ2), and milk fat globule EGF factor 8 (MFGE8). ICAM-1 has been implicated in the regulation of interactions between DCs and T cells [18]. Furthermore, DC-Exos exhibit high expression levels of tetraspanins (CD9, CD37, CD53, CD63, CD81, and CD82), which are involved in various biological processes, including adhesion, fusion, motility, immunological activation, and protein sorting [19]. DC-Exos also contain various endosome-associated proteins like tumor susceptibility gene 101 (Tsg101) and ALG-2-interacting protein X (Alix) (Figure 1). Additionally, DC-Exos contain immunologically active proteins, such as antigen-presenting molecules (MHC-I, MHC-II) and costimulatory molecules, which can directly or indirectly activate immune responses [20]. For instance, DC-Exo carries MHC-I that can bind to tumor-derived peptides, thereby inducing cytotoxic T lymphocytes (CTLs) response and inhibiting tumor growth [21].

DC-Exo membrane is characterized by a typical lipid bilayer structure, comprising sphingomyelin, phosphatidylcholine, phosphatidylethanolamine, phosphatidylinositol, and phosphatidylserine [22]. In comparison to cellular membranes, DC-Exos membranes exhibit elevated concentrations of sphingomyelin and phosphatidylinositol, which contribute to their enhanced stability in biological fluids and under varying pH conditions [23].

In addition, DC-Exos encompass a diverse array of genetic material, including *mRNA*, *miRNA*, *non-coding RNA*, and *DNA*. It has been demonstrated that DC-Exos transmit genetic material between cells, thereby influencing gene expression in recipient cells and modulating intercellular communication [24].

DC-Exos exhibit two distinct phenotypes: mature DC-Exos (mDC-Exos) and immature DC-Exos (imDC-Exos). These two types of exosomes play opposing roles in the immune response, specifically in immunostimulation and immunosuppression. Studies have confirmed that mDC-Exos can activate the immune system, leading to the elimination of tumors or viruses. In contrast, imDC-Exos have the capacity to induce immune tolerance and can be utilized in the treatment of autoimmune diseases. Some studies have also indicated that imDC-Exos may be effective in the treatment of myasthenia gravis [25]. The reason these two types of exosomes exhibit different functions in the immune system is that the proteins, lipids, genetic materials, and other components in mDC-Exos and imDC-Exos differ in their composition. For instance, mDC-Exos contain high levels of MHC-II and costimulatory molecules, while imDC-Exos have lower levels of MHC-II and CD86. Additionally, CD40 and CD80 molecules were not detected [26].

## 3. Methods for Loading Cargo into Exosomes

**Figure 2:**
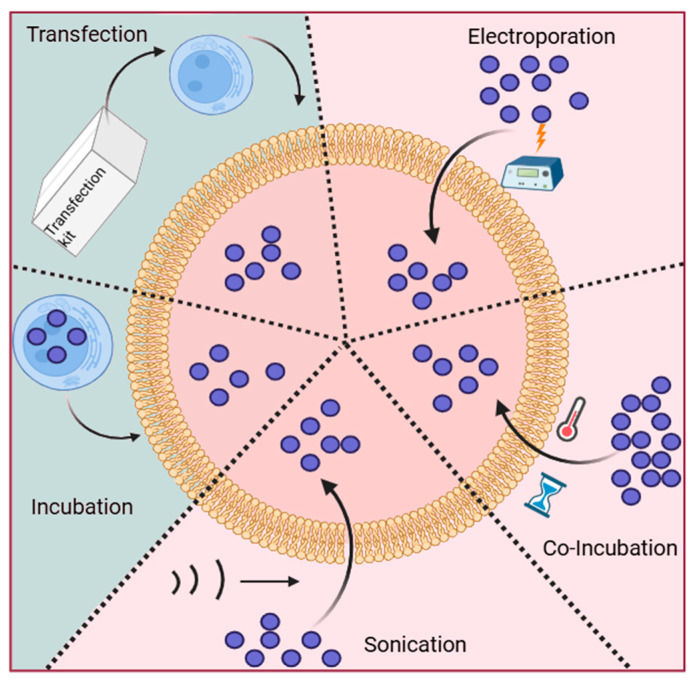
The methods for drug loading into exosomes.

The methods for loading cargo into exosomes are primarily categorized into two types: endogenous loading and exogenous loading (Figure 2 and Table 1) [28]. Exogenous loading involves initially extracting exosomes from specific cells and subsequently introducing drugs into the exosomes via methods like electroporation, co-incubation, sonication, freeze-thaw cycles, and extrusion. In contrast, endogenous loading requires modifications to the exosome-secreting cells. This modification enables cells to incorporate target molecules into the parent cells, which then secrete exosomes containing these target molecules [29].

### 3.1. Exogenous Drug Loading

Exogenous drug delivery methods, such as electroporation, co-incubation, and sonication, are now widely utilized for the delivery of small molecules, proteins, and genes.

The co-incubation method involves mixing isolated exosomes with drugs and incubating the mixture at room temperature. This technique leverages the concentration gradient between the interior and exterior of the exosome membrane, allowing the drug to passively diffuse into the exosome [30]. Additionally, membrane permeabilizers can be added during the incubation process to enhance the rate of drug loading by creating pores on the exosome membrane [31]. Although the co-incubation method is straightforward and easy to implement, causing minimal damage to exosomes, it is primarily suitable for hydrophobic drugs that can interact with the lipid structure of exosomes. In the case of hydrophilic drugs, factors such as pH can be adjusted to facilitate the passage of the drug through the lipid bilayer of the exosome into the hydrophilic core. For instance, some researchers have successfully loaded doxorubicin (Dox) into macrophage-derived exosomes under conditions of pH 8.0 [32].

Electroporation is a technique that employs an electroporator to apply a specific electric field to exosomes and the encapsulated drug. This process induces the formation of pores in the lipid bilayer membrane, thereby facilitating the entry of the drug into the exosome. After drug loading, the exosome must be incubated for a specific duration to allow the pores in the exosome membrane to be restored [33]. Some researchers utilized lung-derived exosomes (Lung-Exo) as a delivery vehicle, loading with *mRNA* and red fluorescent protein (RFP) through electroporation. The findings indicated that *mRNA* and RFP loaded Lung-Exos can be distributed more effectively deeper in the lungs compared to commercial standard biological nanoparticles, HEK293T-derived exosomes (HEK-Exo), and synthetic LNPs (Lipo) [34]. Although electroporation is a faster and less time-consuming method, it has been shown that loading *siRNA* into exosomes via electroporation may result in the aggregation of siRNA [35]. This aggregation can lead to an overestimation of the actual amount of *siRNA* loaded into the exosomes, and the process may also cause damage to the membrane structure of the exosomes [36].

Sonication of exosomes, which involves weakening the lipid bilayer membrane by applying mechanical shear force, creates temporary pores in the exosome membrane. This process facilitates the loading of proteins, drugs, and nucleic acids [37]. Some studies have confirmed that specific sonicated treatments of exosomes demonstrate higher drug-loading efficiency [38]. Currently, the sonication of exosomes for drug loading has been employed in various research studies. For example, Dulla et al. employed sonication to incorporate paclitaxel and 5-fluorouracil into bovine milk exosomes, aiming to enhance the bioavailability and reduce the toxicity of these drugs [39]. Although sonication offers advantages such as high drug-carrying efficiency and sustained drug release, it can also cause damage to the exosome membrane, potentially compromising its integrity and leading to issues like exosome aggregation [40]. Nevertheless, sonication remains the predominant method for exosome DDS and holds significant potential for optimizing drug delivery [41].

In addition to co-incubation, electroporation, sonication, extrusion, freeze-thawing, and saponin treatment can be employed for loading drugs into exosomes. For instance, in the extrusion method, exosomes and the drug are mixed homogeneously and then repeatedly extruded through an extruder with a pore size of 100–400 nm [42]. The advantage of this method is that it yields exosomes with a uniform particle size distribution and a relatively high drug loading efficiency. However, repeated extrusion may lead to alterations in the membrane structure of the exosomes [43].

### 3.2. Endogenous Drug Loading

Endogenous loading refers to the process by which cargo is incorporated during exosome biosynthesis. This means that the cargo is either loaded directly into the parental cells or achieved through genetic modifications that enable the parental cells to secrete engineered exosomes [44]. There are two primary methods of endogenous loading: incubation and genetic modification.

Endogenous incubation differs from exogenous incubation in that it involves the co-incubation of the loaded cargo with the parental cells. In this process, the loaded cargo passes through the cell membrane into the cell, and the cytoplasmic cargo is subsequently released from the cell along with the exosome, resulting in the formation of an exosome that is loaded with therapeutic cargo [44]. For example, Luisa et al. co-incubated paclitaxel with mesenchymal stem cells (MSCs) for 24 h to obtain paclitaxel-loaded MSCs, from which they subsequently collected paclitaxel-loaded exosomes from the cell culture medium [45]. Although several studies have demonstrated that this method can produce exosomes loaded with effective therapeutic cargo, it requires that the therapeutic cargo be non-toxic to the parental cells, which limits the generalizability of the method.

Genetic modification involves the overexpression of a desired therapeutic agent achieved through the transfection and alteration of the parental cells’ genetic material [46]. Transfection refers to the process of introducing specific plasmids into cells through the use of particular reagents, thereby facilitating the ectopic expression of target proteins, peptides, or nucleic acids. These molecules are subsequently incorporated into exosomes during the biosynthesis of exosomes within the cell. miRNAs can be efficiently and safely loaded into exosomes, and they are currently being utilized in various research studies [47]. For instance, Lou et al. transfected adipose tissue-derived mesenchymal stem cells (AMSCs) with a *miR-122* expression plasmid. Following a 48 h transfection period, exosomes derived from AMSC were isolated and subsequently introduced to recipient hepatocellular carcinoma (HCC) cells. The findings indicated that *miR-122*-transfected AMSCs were capable of effectively producing exosomes that contained *miR-122* [48]. Kojima et al. transfected HEK-293T with a specifically designed plasmid to produce exosomes containing catalase *mRNA* [49]. Overall, transfection is considered a relatively safe and effective technique for loading therapeutic agents into exosomes. However, some studies have indicated potential risks, including contamination of parental cells and exosomes by transfection reagents, as well as the possibility of hemolytic toxicity [50].

In addition to these two methods, electroporation can also be utilized for endogenous drug loading. For example, Ma et al. electroporated human adipose-derived mesenchymal stem cells (hAdMSCs) using a track-etched membrane. They loaded a mixture of human vascular endothelial growth factor A (VEGF-A) and human bone morphogenetic protein 2 (BMP-2) *mRNAs* into the hAdMSCs, prompting them to secrete exosomes that carried both VEGF-A and BMP-2 *mRNAs*. These exosomes were then used to treat rats with critical-size defects [51].

**Table 1:** The exosome source and delivery cargo of the exosome loading methods.

	Loading Methodology	Exosome Source	Delivery Cargo	References
Exogenous Drug Loading	Co-incubation	Dendritic cells, milk, human monocytic cells (THP-1 cell line)	Triptolide, curcumin, berry anthocyanidins, doxorubicin, cholesterol-modifed *microRNA 159*	[27,52,53,54]
	Electroporation	Dendritic cells	*siRNA*, *BRAF siRNA*, doxorubicin	[55,56,57]
	Sonication	Macrophage, bovine milk	Paclitaxel, 5-fluorouracil	[39,58]
Endogenous Drug Loading	Endogenous incubation	Macrophages, mesenchymal stromal cells	Curcumin, paclitaxel	[45,59]
	Transfection	Dendritic cells, mesenchymal stem cells, HEK-293 T cells	Lamp2b protein, *miR-125b*, *microRNA-122*, catalase *mRNA*	[10,48,49,60]

## 4. Types of Drug Loading for DC-Exos

Alvarez et al. utilized DC-Exo as a carrier to load exogenous *siRNAs* via electroporation for delivery to the mouse brain. The findings of this study provide compelling evidence that DC-Exo can serve as effective drug delivery carriers [10]. Currently, DC-Exo has been utilized in various drug delivery studies. DC-Exo is capable of transporting a diverse array of cargoes, which include small molecules (such as paclitaxel, curcumin, and doxorubicin), genetic materials (including *microRNAs* and *siRNAs*), and proteins (such as enzymes, transmembrane proteins, and cytoskeletal proteins) [50].

**Figure 3:**
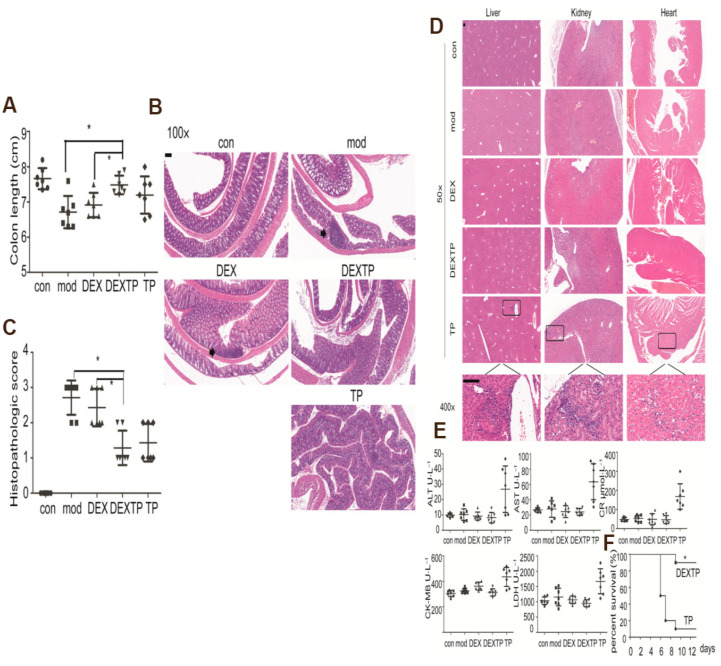
(**A**) Colon length of mice in each group (* *p*  <  0.05). (**B**) H&E staining showed that DC-EXOTP (DEXTP) reduced mucosal damage and inflammatory cell infiltration in the colon (the scale bar represents 100 μm). Arrowheads point to a microabscess and inflammatory cell infiltration. (**C**) Histological colitis score of mice in each group (two-tailed *t* test, * *p*  <  0.05). (**D**) H&E staining of liver, kidney, and heart tissues from each group. Enlarged view showed pathological damage to organs in the TP group. No obvious injury was observed in DEXTP and other groups (the scale bar represents 100 μm). (**E**) Serum biochemical indicators including ALT, AST, Cr, CK-MB, and LDH were detected and showed liver, kidney, and heart injury in some of the mice in the TP group. There is no significant difference among the other groups. (**F**) Survival time of mice from increased dose groups were recorded (*n*  =  10, * *p*  <  0.05) [27].

Numerous small-molecule drugs, including paclitaxel, triptolide, curcumin, and other drugs, demonstrate significant therapeutic efficacy on malignant tumors. However, the clinical application of these drugs often fails to achieve the expected therapeutic effects [61]. Consequently, the identification of an effective delivery vehicle that can mitigate the negative effects of these drugs while preserving their therapeutic activity has become a central focus and challenge in contemporary research. DC-Exo possesses a lipid bilayer structure that confers resistance to degradation by enzymes and other reagents. Consequently, the cargo contained within these exosomes exhibits high stability. Additionally, exosomes demonstrate low immunogenicity and high biocompatibility, rendering them suitable as delivery carriers for small-molecule drugs [41]. The delivery of small molecule chemotherapeutic agents through DC-Exo has been documented in numerous studies [62]. Triptolide, identified as the most potent active compound in Tripterygium wilfordii Hook F (TwHF) extracts, has demonstrated significant immunosuppressive properties and anti-inflammatory effects in the context of autoimmune diseases and transplant rejection. Conversely, triptolide is associated with severe toxic side effects affecting multiple organs, which considerably restricts its clinical applicability [63]. Rao et al. have employed DC-Exo as a vehicle for triptolide in the treatment of colitis and rheumatoid arthritis in murine models. The findings indicated that DC-Exo effectively delivered triptolide to DCs and produced therapeutic effects. Notably, no toxicity associated with triptolide was observed in the mice treated with DC-Exo-loaded triptolide, thereby presenting a novel strategy to mitigate the toxic side effects of triptolide (Figure 3) [27]. Furthermore, these results suggest that DC-Exo may serve as an optimal vehicle for drug delivery.

**Figure 4:**
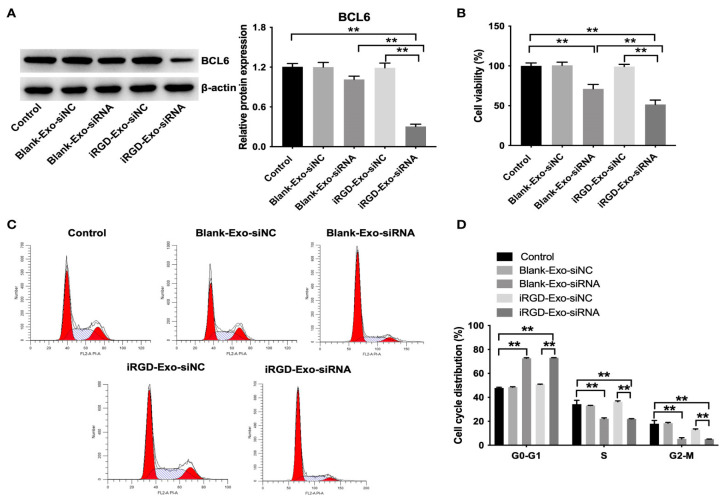
In vitro antitumor effect of iRGD-Exos-BCL6 siRNA. (**A**) Western blot analysis of BCL6 expressions in OCI-Ly8 cells. (**B**) CCK-8 assay was used to detect cell proliferation. (**C**,**D**) Flow cytometry assay was used to detected cell cycle distribution. ** *p* < 0.01. All tests were repeated in triplicate [55].

**Figure 5:**
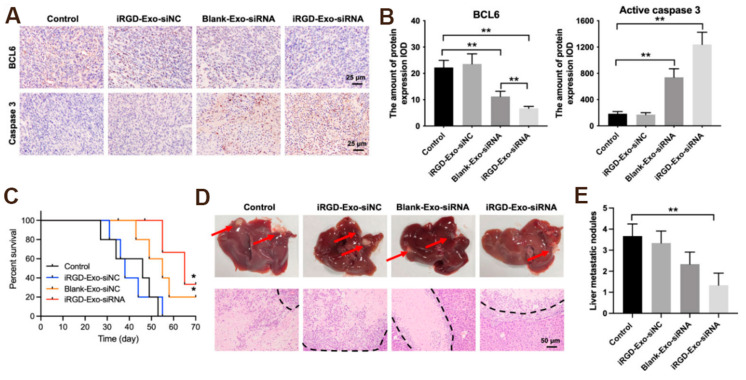
Tissue analyses. (**A**,**B**) IHC analysis of BCL6 and active caspase 3 expressions in tumor tissues collected from nude mice injected with OCI-Ly8 cells and exosomes via tail vein (scale bar, 25 μm). IOD, integrated optical density. (**C**) The survival rate of mice is shown. (**D**,**E**) Representative images of tumor burdened liver tissues and their HE-stained sections. Red arrows, metastasis nodules in liver tissues. White section, tumor nodules. * *p* < 0.05, ** *p* < 0.01 [55].

DC-Exo functions as a delivery vehicle for various types of genetic materials, including *small interfering RNA* (*siRNA*), *microRNA* (*miRNA*), *messenger RNA* (*mRNA*), *circular RNA*, and *spherical nucleic acids* (*SNA*) [64]. Gene therapy utilizing nucleic acid substances, such as *DNA* and *RNA*, represents a promising approach for the treatment of cancer. *Small RNAs* such as *miRNAs* and *siRNAs* have the capacity to bind to *mRNAs* within target cells, thereby regulating gene expression [65]. However, most of these nucleic acid substances exhibit high hydrophilicity and possess a negative charge, which poses significant challenges to their ability to traverse cell membranes and enter cells. Additionally, the presence of various nucleases, along with hepatic and renal clearance mechanisms, contributes to the degradation or elimination of these nucleic acid substances prior to cellular uptake. The inherent instability and degradability of nucleic acid substances substantially restrict their application in therapeutic contexts [41]. Consequently, the utilization of DC-Exos as carriers for the delivery of nucleic acid drugs emerges as a promising strategy for enhancing their therapeutic efficacy. *miRNAs* are a class of non-coding *RNAs* consisting of 20 to 24 nucleotides that play a crucial role in the regulation of gene expression [66]. To efficiently deliver effective *miRNAs* to target cells for therapeutic purposes and to enhance the stability and degradability of *miRNAs*, numerous studies have investigated the utilization of DC-Exo as a vehicle for *miRNA* delivery to target cells. Yin et al. demonstrated that DC-Exo overexpressing *microRNA-146a* significantly inhibited the progression of autoimmune severe myasthenia gravis in experimental mice [25]. *siRNA* can bind to complementary sequences in the *mRNA* of target cells, thereby inhibiting gene expression through the degradation of the bound *mRNA* [66]. However, *siRNAs* exhibit instability and are prone to degradation during the systemic circulation. DC-Exo can serve as an effective delivery vector to mitigate this degradation and facilitate the targeted delivery of *siRNAs* to specific cells [67]. Some researchers utilized imDC-Exo with low immunogenicity to deliver BCL6 *siRNA* to tumor tissues. The findings of the study demonstrated that DC-Exo encapsulating BCL6 *siRNA* effectively inhibited the proliferation of diffuse large B-cell lymphoma (DLBCL) in vitro and significantly suppressed tumor growth in murine models without exhibiting notable toxicity (Figure 4 and Figure 5) [55]. In conclusion, the results of this study suggest that DC-Exo possesses considerable therapeutic potential as a vehicle for *siRNA* delivery.

Protein-based therapeutics primarily encompass antibody drugs, enzymes, peptide hormones, and blood-derived products [68]. In recent years, protein-based therapeutics have garnered significant attention for their efficacy, specificity, and low toxicity in the treatment of various diseases. However, these protein drugs often exhibit considerable immunogenicity, which can lead to a range of immune and allergic reactions within the body [41]. Furthermore, the challenges associated with the safe and effective delivery of protein therapeutics are compounded by their inherent instability in the blood circulation, susceptibility to enzymatic degradation, short half-life, and low transmembrane transport rates [69]. The enhancement of delivery efficiency for protein therapeutics, as well as the prevention of their degradation and denaturation, represents a pressing challenge that necessitates resolution. DC-Exo exhibits considerable potential for the delivery of biomolecular drugs, attributable to their inherent capacity to transport bioactive molecules, their proficiency in traversing biological barriers, and their low immunogenicity [44]. Although research on the application of DC-Exo for the delivery of protein drugs remains limited, investigations have demonstrated the use of DC-Exo to facilitate the delivery of chaperone-rich cell lysate (CRCL) derived from GL261 glioma cells, which was subsequently loaded into DCs for glioma treatment. In vivo, studies indicated that DC-EXO (CRCL-GL261)-DCs significantly extended survival time and inhibited tumor growth in mice bearing gliomas [70]. This research offers a novel direction and conceptual framework for the development of CRCL-based anti-tumor vaccines.

## 5. Surface Engineering Modifications of DC-Exos

Certain engineering modifications to the surface of exosomes can significantly expand their applicability in DDS. Presently, exosomes can be endowed with specific properties through various modification techniques. In comparison to natural exosomes, modified exosomes may exhibit enhanced capabilities such as active targeting, pH sensitivity, and prolonged circulation. Research has demonstrated that specific modification of exosomes can substantially improve their utility in therapeutic applications within the realm of nanomedicine.

### 5.1. Targeted Modifications

**Figure 6:**
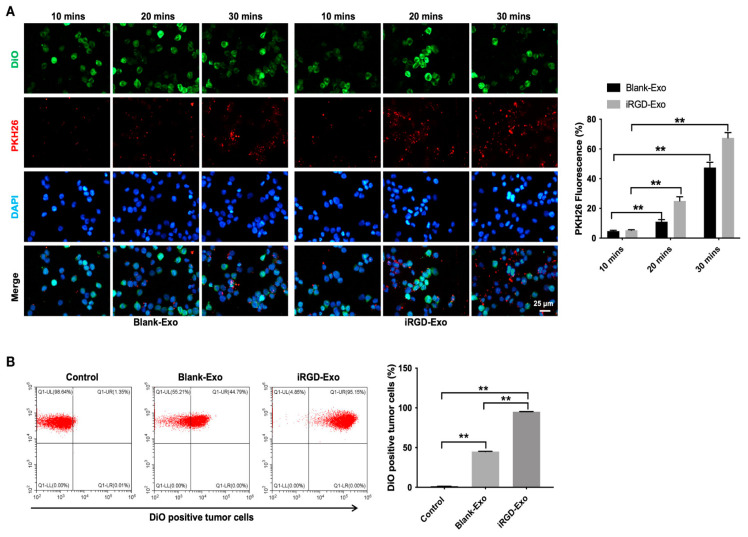
Targeting DLBCL cells via iRGD-Exo. (**A**) Confocal microscopy images of colocalization of exosomes in OCI-Ly8 cells. The nucleus was stained with DAPI (blue), the cell membrane was stained with DiO (green), and exosomes were labeled with PKH26 (red). Scale bar, 25 μm. (**B**) Flow cytometry analysis of the proportion of OCI-Ly8 cells that were bound to iRGD-Exo. ** *p* < 0.01. All tests were repeated in triplicate [55].

Although DC-Exo exhibits a certain homing effect and can naturally target DCs, their accumulation in target organs that are protected by physiological barriers is limited [71]. Furthermore, the uptake capacity of DC-Exo varies between different cell types [72]. Therefore, to enhance the delivery of DC-Exo and their encapsulated therapeutic agents to specific tissues or cells, it is necessary to implement certain modifications to the DC-Exo. For instance, certain researchers have transfected DC to secrete iRGD-exosomes, which are exosomes that express the iRGD peptide. This peptide has the capability to specifically bind to OCI-Ly8 cells, which are human diffuse large B-cell lymphoma cells characterized by high expression of the avβ3 integrin. Consequently, this modification enhances the targeting efficiency of DC-Exo towards tumor cells (Figure 6) [55]. In addition, Alvarez-Erviti et al. obtained DC-Exos expressing the Lamp2b protein through transfection modification, which can bind to neuron-specific RVG peptides and load siRNA via electroporation, thereby facilitating the targeted delivery of siRNA [10].

### 5.2. pH Sensitivity and Long-Circulating Modifications

**Figure 7:**
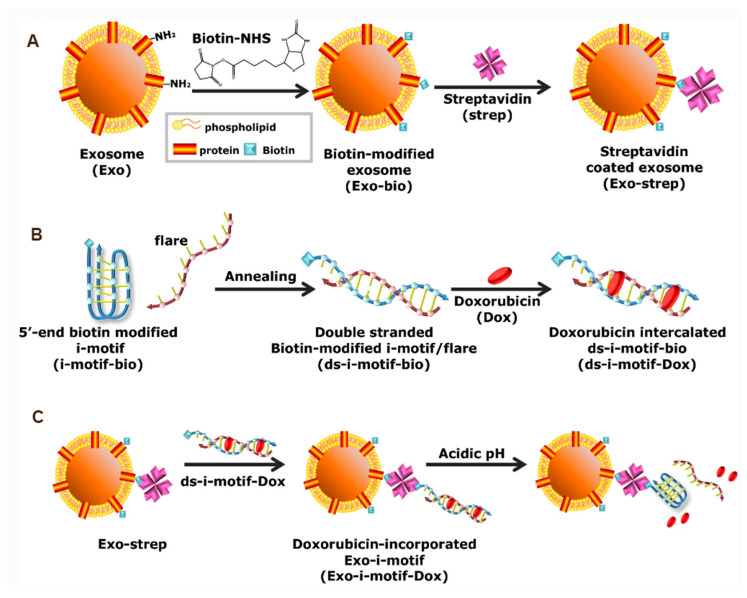
Schematic illustration of (**A**) preparation of biotin-modified exosomes (Exo-bio) and streptavidin-coated exosomes (Exo-strep). (**B**) Intercalation of Dox within double-stranded DNA composed of the biotin-modified I-motif (i-motif-bio) and flare (ds-i-motif-bio). (**C**) Complexation of Exo-bio-strep-ds-i-motif-bio (Exo-i-motif) and incorporation of Dox for pH-responsive delivery of Dox [73].

Tumor microenvironments have been observed to exhibit acidity in comparison to normal tissues. Consequently, research has been undertaken to improve the delivery of therapeutic agents to tumor sites by developing pH-responsive DDS tailored for the acidic conditions of tumors. The i-motif, a pH-responsive DNA strand rich in cytosine, has been employed to enhance the tumor site release of Dox through the delivery of i-motif-modified exosomes (Figure 7) [73]. AA exhibits a specific binding affinity for the sigma receptor, which is prominently expressed on the surface of tumor cells. Several studies have investigated the use of AA-PEG-modified exosomes encapsulating paclitaxel for the treatment of pulmonary metastases. These studies have demonstrated that AA-PEG-modified exosomes possess notable tumor-targeting properties and exhibit reduced recognition and internalization by the mononuclear phagocyte system (MPS) due to the PEG modification, thereby extending their circulation time in vivo [58]. However, the aforementioned modifications have not yet been implemented in DC-Exo modification, and further research and development are required to explore their potential applications in this context.

## 6. Progress in the Utilization of DC-Exos as Drug Delivery Vehicles

The utilization of exosomes, particularly DC-Exo as DDS presents several advantages compared to traditional methods. Like DCs, DC-Exo exhibits a diverse array of immune-related molecules on their membrane surface, which play a crucial role in antigen presentation and the activation of CD4+ and CD8+ T cells [74]. It has been demonstrated that DC-Exo can stimulate cytotoxic T cells and inhibit tumor growth when utilized as a drug carrier in tumor therapy. Furthermore, DC-Exo can function as a cell-free vaccine for anticancer immunotherapy. Compared to DC vaccines, DC-Exo vaccines exhibit greater stability [75] and may elicit a more robust stimulatory effect on T cells [76]. DC-Exo not only acts as a natural carrier for therapeutic agents but also possesses the capability to activate the body’s immune response. This dual functionality has garnered significant attention and has been applied in therapeutic research across a variety of diseases.

### 6.1. DC-Exos as Drug Delivery Carriers in Cancer Therapy

Cancer remains a significant public health challenge worldwide, with 19.3 million new cases and nearly 10 million cancer-related deaths reported in 2020, making it the leading cause of mortality [77]. Presently, the primary treatment modalities for cancer encompass surgery, radiotherapy, chemotherapy, and immunotherapy [78]. Among these, surgery is applicable for the management of most solid tumors [79]; however, the presence of residual tumor cells necessitates the use of adjuvant chemotherapy and radiotherapy. Chemotherapy remains the most effective and cost-efficient treatment option for cancer; however, many chemotherapeutic agents are associated with significant adverse effects. Traditional chemotherapeutic drugs often lack sufficient specificity for cancer cells [80] and can also adversely affect normal cells [81]. Consequently, addressing the challenges of reducing the toxicity and side effects of these therapeutic agents, enhancing their bioavailability, and minimizing their impact on normal cells and tissues is a pressing concern in contemporary cancer research.

**Figure 8:**
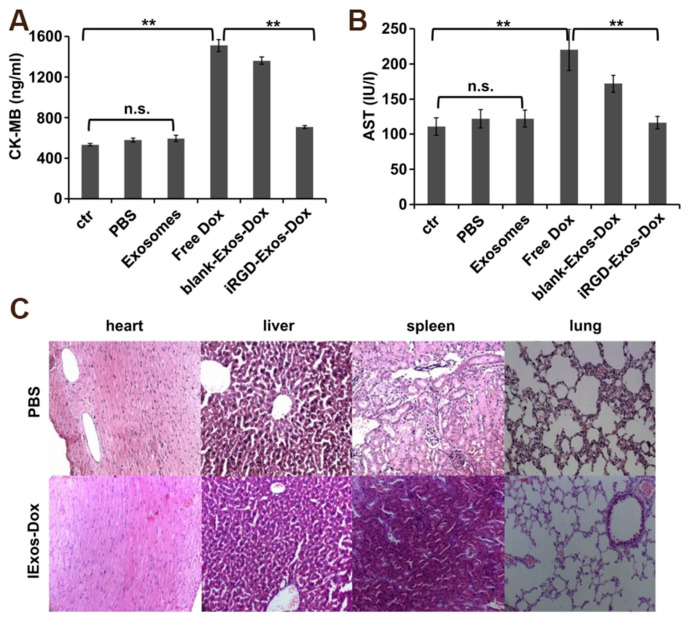
Evaluation of the toxicity profile of iRGD-Exos-Dox. (**A**,**B**) Serum markers of cardiac damage. Serum was collected from tumor-bearing mice treated with six injections of PBS, iRGD-Exos, Free Dox (3 mg/kg), or an equivalent amount of Dox incorporated into blank-Exos (blank-Exos-Dox) or iRGD-Exos (iRGD-Exos-Dox), and the activities of CK-MB (**A**) and AST (https://www.sciencedirect.com/topics/biochemistry-genetics-and-molecular-biology/aspartate-transaminase) (accessed on 27 January 2025). (**B**) were measured. Each bar represents mean ± SD of three replicates. n.s., not significant; **, *p* < 0.01. (**C**) Various organs from mice treated as described in (**A**,**B**) were stained with H&E. No overt tissue damage was observed in iRGD-Exos-Dox treated mice [57].

Currently, exosomes derived from DCs are being utilized as drug-delivery vehicles in cancer therapy. For instance, Lin et al. employed mDC-Exos, which possess immune-inducing properties, as carriers for the delivery of *siRNA* (*siBRAF*) aimed at silencing gene mutations associated with malignant melanoma (MM). This study demonstrated that *siBRAF*-mDC-Exos exhibited enhanced BRAF gene silencing efficacy compared to free *siBRAF*, resulting in increased anti-MM activity and a high safety profile in both in vivo and in vitro settings [56]. Xu et al. conducted a study in which they loaded fluorouracil into DC-Exo for the purpose of researching and treating colon cancer. The findings indicated that DC-Exo not only inhibits tumor growth but also enhances the anti-colon cancer efficacy of fluorouracil when utilized as a carrier [82]. To mitigate immunogenicity and toxicity, certain researchers have employed imDC-Exo loaded with Dox in the context of breast cancer research and treatment. The study demonstrated that exosomes loaded with Dox, specifically those produced from immature DCs (iRGD-Exos-Dox), exhibited reduced cardiotoxicity in comparison to free Dox. Furthermore, no significant tissue damage was observed in mice treated with iRGD-Exos-Dox when compared to control groups (Figure 8). This investigation substantiates the assertion that DC-Exo serves as a safe and effective drug delivery vehicle for targeted tumor therapy [57].

### 6.2. DC-Exos as Drug Delivery Carriers in the Treatment of Immune Diseases

In addition to the immunostimulatory capabilities of DCs, DC-Exo has been reported to play a role in various immune processes, including antigen presentation, immunomodulation, and signaling. Consequently, numerous researchers have begun to utilize DC-Exo in the treatment of various immune-related diseases [83]. Rao et al. utilized DC-Exo as a carrier for the delivery of triptolide (TP) in the treatment of autoimmune diseases, specifically ulcerative colitis (UC) and rheumatoid arthritis (RA). The findings indicated that DC-EXOTP was effective in reducing inflammatory cell infiltration, mitigating colonic mucosal damage, and decreasing the incidence of crypt abscesses in murine models of colitis. The experimental data demonstrated that DC-EXOTP significantly ameliorated colonic inflammation and effectively diminished local colonic damage in mice with induced colitis. Furthermore, in comparison to TP, DC-EXOTP exhibited no significant adverse effects on several organs, including the liver, heart, and kidneys. Additionally, the study confirmed the efficacy of DC-EXOTP in alleviating RA without significant toxicity. In summary, DC-EXOTP not only substantially alleviated symptoms of UC and RA, but also effectively reduced the toxicity associated with TP [27].

Other researchers have utilized DC-Exo in conjunction with the immuno-regulatory cytokines TGFB-1 and IL-10 for the treatment of experimental degenerative bone disease. DC-Exo has been shown to protect against clearance and proteolytic degradation, thereby promoting the accumulation of TGFB-1 and IL-10 at the site of the disease. Both in vitro and in vivo studies have demonstrated that DC-Exo encapsulating TGFB-1 and IL-10 inhibits DC maturation, reduces local Th17 effects, and enhances the recruitment of T-regulatory cells (T-regs). These mechanisms collectively contribute to the inhibition of bone-resorptive cytokines and a reduction in osteoclastic bone loss. This study confirms the feasibility of using DC-Exo as a delivery vehicle in the treatment of degenerative bone disease and may offer additional therapeutic strategies for managing this condition [84].

### 6.3. The Utilization of DC-Exos as Cell-Free Vaccines

DCs, recognized as the most potent antigen-presenting cells (APCs), are integral to the regulation and initiation of both innate and adaptive immune responses [85]. These cells are capable of activating naive antigen-specific CD4 and CD8 T cells through the uptake, processing, and presentation of antigens, thereby initiating an adaptive immune response within the organism [86]. Furthermore, DCs can present processed exogenous antigenic peptides to CD8 T cells via MHC-I, a process referred to as cross-presentation [87]. Research has demonstrated that DCs play a significant role in the induction of anti-tumor CD8 T cell immunity and in mediating CD8 T cell tolerance [88]. Consequently, DC vaccines, which leverage the capacity of DCs to enhance the host’s ability to combat tumors, have emerged as a primary strategy in cancer immunotherapy. Despite their potential, DC vaccines derived from monocytes exhibit considerable limitations, as evidenced by the fact that only 5% to 15% of patients in clinical trials demonstrate objective immune responses [89]. One of the primary factors contributing to the limited efficacy of DC vaccines is tumor-mediated immunosuppression. This phenomenon involves the presence of various immunosuppressive factors within the tumor microenvironment, which can function as immunosuppressants, thereby inhibiting the clinical effectiveness of DC vaccines by modulating the immune response of anti-tumor T cells [90]. Furthermore, DC vaccines face several challenges, including high preparation costs, a short shelf-life, difficulties in storage, and issues related to production standardization [75]. In contrast, DC-Exo exhibits greater circulating stability compared to DCs themselves, and DC-Exo is enriched in peptide-MHC-II complexes at levels 10 to 100 times higher than those found in DCs [20]. Furthermore, cell-free DC-Exo does not respond to immunosuppressive molecules to the same extent as DC vaccines, which may result in enhanced immunostimulatory effects on T cells [21]. Consequently, DC-Exo-based vaccines demonstrate significant potential in the realm of cancer immunotherapy and have emerged as promising cell-free therapeutic vaccines, garnering considerable interest from an increasing number of researchers.

**Figure 9:**
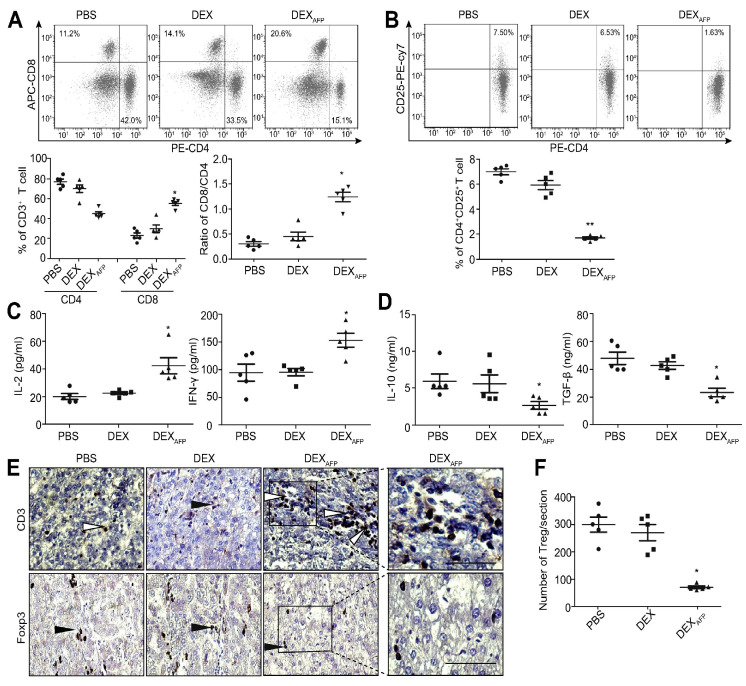
DC-EXOAFP (DEXAFP) improved the tumor microenvironment (https://www.sciencedirect.com/topics/pharmacology-toxicology-and-pharmaceutical-science/tumor-microenvironment) (accessed on 27 January 2025) in DENA-induced autochthonous HCC mice. (**A**) Analysis of CD8CD3 (https://www.sciencedirect.com/topics/immunology-and-microbiology/cd3-antigen) (accessed on 27 January 2025) and CD4CD3 T lymphocytes in tumor tissues from treated tumor-bearing mice. (**B**) Flow cytometry analysis of CD25CD4 (https://www.sciencedirect.com/topics/biochemistry-genetics-and-molecular-biology/cd25) (accessed on 27 January 2025) Treg (https://www.sciencedirect.com/topics/immunology-and-microbiology/regulatory-t-cell) (accessed on 27 January 2025) cells in tumor tissues from treated tumor-bearing mice. (**C**) Measurement of IFN-γ and IL-2 in tumor tissues from treated tumor-bearing mice on week 40 after induction. (**D**) Measurement of TGF-β and IL-10 in tumor tissues from treated mice with ELISA. (**E**) Immunohistochemistry (https://www.sciencedirect.com/topics/medicine-and-dentistry/immunohistochemistry) (accessed on 27 January 2025) of CD3 and FoxP3 Treg cells in tumor sections from treated autochthonous HCC mice (scale bar = 100 μm). (**F**) Quantitative analysis (https://www.sciencedirect.com/topics/biochemistry-genetics-and-molecular-biology/quantitative-technique) (accessed on 27 January 2025) of FoxP3 Treg cells in tumor tissues from treated autochthonous HCC mice. Significance was determined with two-tailed *t* test (* *p* < 0.05; ** *p* < 0.01) [93].

Currently, numerous researchers have employed DC-Exo vaccines in the investigation and treatment of various tumors. HCC has emerged as one of the most lethal malignant tumors globally, attributable to its aggressive nature, high mortality rate, and poor response to conventional clinical treatments [91]. While chemotherapy and radiotherapy are commonly utilized in the clinical management of HCC, their benefits in terms of survival are limited. Surgical resection can be effective for early-stage HCC; however, this approach is applicable only to a subset of patients and is associated with a high likelihood of recurrence following the procedure [92]. Zhen et al. utilized a cell-free vaccine comprising HCC α-fetoprotein (AFP) modified DC-Exo in their investigation of HCC. The findings of this study indicated that DC-ExoAFP was capable of eliciting a robust antigen-specific immune response and demonstrated superior inhibition of tumor growth in various mouse models, including ectopic, orthotopic, and carcinogen-induced HCC. Furthermore, this study found that treatment with DC-ExoAFP transformed the tumor microenvironment in mice from an immunosuppressive state to an immunostimulatory state (Figure 9). However, the study did not demonstrate significant efficacy of DC-ExoAFP when administered to athymic nude mice and CD8 T cell-depleted mouse HCC tumor models. This observation leads to the speculation that T cells may play a crucial role in mediating the anti-tumor effects of DC-ExoAFP [93]. A separate investigation examined the combination of the DC-Exo vaccine and microwave ablation (MWA) in the context of anti-tumor research for HCC. The findings indicated that the tumor-suppressive efficacy of the combined treatment with MWA and DC-EXO vaccine was significantly superior to that of the MWA treatment alone. Additionally, flow cytometry analysis revealed a reduction in the proportion of splenic Treg in the group receiving the combined treatment compared to the MWA-only group. In conclusion, this study suggests that the therapeutic effects of the DC-Exo vaccine and DC vaccine are comparable when utilized in combination therapy [94]. A recent study conducted by another researcher developed a novel anti-tumor nano-vaccine by encapsulating neoantigens within DC-Exo. The findings indicated that this nano-vaccine significantly inhibited tumor growth in comparison to free neoantigens and exhibited a notable preventive effect on cancer occurrence. This study also confirms that the proteins present on the surface of DC-Exo may contribute to its anti-tumor effects. This innovative nano-vaccine establishes a foundation for future research on DC-Exo-based vaccines and their clinical applications while also broadening the therapeutic potential of DC-Exo [95].

### 6.4. DC-Exos in Combination with Toxoplasma Gondii

**Figure 10:**
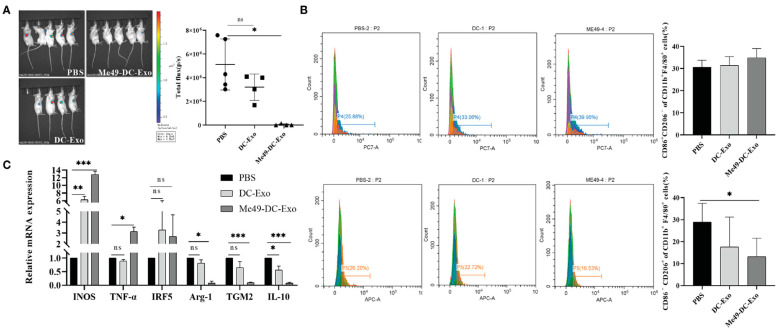
Me49-DC-Exo inhibited tumor growth in mice and regulated macrophage polarization. (**A**) On Day 4 after treatment, the IVIS imager detected bioluminescence images in tumors of mouse and quantified the bioluminescence signal intensity of each tumor in mouse. (**B**) Flow cytometry was used to label CD86 + or CD206 +, and CD45 + CD11b + F4/80 + macrophages in blood of mice injected with DC-Exo and Me49-DC-Exo were stained to detect the percentage of CD86+ CD206 − M1 macrophages and CD86 − CD206 + M2 macrophages. (**C**) mRNA levels of M1 macrophage specific genes and M2 macrophage specific genes in blood of tumor-bearing mice injected intratumorally with PBS, DC-Exo, and Me49-DC-Exo were detected by qRT-PCR. n.s., not significant (*p* ≥ 0.05), * *p* < 0.05, ** *p* < 0.01, *** *p* < 0.001 [97].

Toxoplasma gondii is a eukaryotic parasite capable of infecting both humans and animals. Research has indicated that infections with Toxoplasma gondii may exert an inhibitory effect on tumor growth [96]; however, the direct application of live Toxoplasma gondii for treatment raises significant safety and ethical concerns. Consequently, there is a pressing need to develop a safer and more rational immunotherapy strategy for tumors that is based on Toxoplasma gondii. Some researchers infected DCs with the Toxoplasma gondii Me49 strain and isolated exosomes (Me49-DC-Exo) from infected DCs. Their results confirmed that Me49-DC-Exo significantly inhibited the growth of colorectal cancer and reduced the proportion of M2 macrophages in mice in vivo compared to DC-Exos from uninfected DCs (Figure 10A–C) [97]. Another study obtained (Tg-DC-Exo) from DCs infected with Toxoplasma gondii RH strain was used to study its anti-infection effect, and it was found that the expression of *miR-155-5P* in Tg-DC-Exo was upregulated, and the infection signal was delivered to the RAW264.7 cells through Tg-DC-Exo, which resulted in the inhibition of Toxoplasma gondii proliferation in RAW264.7 cells [98]. In addition, Aline et al. demonstrated that DC-Exos pulsed with Toxoplasma gondii antigens (TAgs) triggered strong antigen-specific cellular and humoral immune responses in vivo, suggesting the potential application of TAgs-pulsed DC-Exos in the immunoprophylactic treatment of Toxoplasma gondii [99]. In conclusion, the association of DC-Exo with Toxoplasma gondii not only reduced the safety concerns of Toxoplasma gondii but also expanded the application of DC-Exo as a drug delivery carrier.

## 7. Conclusions and Prospects

Exosomes secreted by DCs have demonstrated significant potential in DDS due to their advantageous drug-carrying capabilities and unique immunological functions. Nevertheless, several challenges remain in the clinical application of DC-Exos-based delivery systems, including problems regarding the purity and separation of DC-Exos, the requirement for standardization and quality control in large-scale production, high production costs, instability during long-term storage, and limited drug-carrying capacity, which hinder the further progress of DC-Exo applications [50,100]. Lastly, the immunogenicity of DC-exos remains abstruse. For instance, macrophages and other immune cells may phagocytose and clear DC-exos, thus reducing their circulation time and impeding the efficiency of drug delivery. Additionally, repeated administration of DC-exos may lead to the generation of antibodies against the surface antigens of exosomes. These antibodies can bind to DC-exos, forming immune complexes. This not only impairs the functionality of DC-exos but also has the potential to induce adverse reactions, such as allergies.

Clinical trials involving DC-Exos are still in the early stages. Phase I and Phase II clinical trials have shown that DC-Exos are well-tolerated and safe, with some patients achieving disease stabilization (Table 2). However, only partial or mild therapeutic responses have been observed in treated patients [101,102]. Certain engineering modifications to DC-Exos can significantly enhance drug utilization and reduce side effects. However, further clinical applications of DC-Exos-based engineering modifications still face significant challenges. For instance, DC-Exos exhibit low yields and low drug loading efficiency. Although various bioengineering strategies have been employed to enhance the production of DC-Exos, these efforts have introduced additional challenges in the production process, including potential byproducts and side effects associated with the bioengineering techniques. Therefore, there is a pressing need to develop methodologies for the standardization and validation of products derived from DC-Exos [103]. Secondly, during the engineering of DC-Exos, some damage may occur to the membrane of the DC-Exos, thereby impairing their natural targeting and delivery properties [104]. In addition, the inherent complexity of engineered modifications, along with their unpredictability and uncertainty, makes the regulatory approval process for DC-Exos somewhat challenging [105].

In summary, the exploration of DC-Exos-based therapies is still in its nascent stages, and the intricate immunotherapeutic mechanisms underlying DC-Exos warrant further investigation to uncover additional therapeutic possibilities.

## Figures and Tables

**Table 2:** Summary of current clinical trials with DC-Exos.

Trial ID	Cancer Type	Phase	Exosomes/Antigen	Doses	References
TrialTroveID-033370	Advanced non-small-cell lung cancer	I	Exosomes were isolated from autologous MoDCs generated in vitro, and loaded with MAGE peptides.	Once weekly for 4 weeks	[101,106]
TrialTroveID-033368	MAGE3- expressing advanced melanoma	I	Autologous MoDC-derived exosomes loaded with MAGE3 peptides.	Once weekly for 4 weeks	[17,107,108]
TrialTroveID-084015	Advanced colorectal cancer	I	Exosomes from patient ascites + GM-CSF, ASexos contained CEA with no additional antigen loading.	Once weekly for 4 weeks	[109,110]
NCT01159288 TrialTroveID-130822	Non-small-cell lung cancer	II	IFN-γ-matured autologous MoDCs loaded with both MHCI and MHCII tumor epitopes.	Exosome immunization in 1-, 2-, and 3-week intervals in a maintenance immunotherapy regime	[111,112]

## Abbreviations

DC-Exos	Dendritic-cell-derived exosomes	DDS	Drug delivery system
PEG	Polyethylene glycol	RES	Reticuloendothelial system
ICAM-1	Intercellular adhesion molecule 1	αMβ2	Integrin α and β-chains
MFGE8	Milk fat globule EGF factor 8	Tsg101	Tumor susceptibility gene 101
Alix	ALG-2-interacting protein X	CTLs	cytotoxic T lymphocytes
mDC-Exos	Mature DC-Exos	imDC-Exos	Immature DC-Exos
Dox	Doxorubicin	MSCs	Mesenchymal stem cells
AMSCs	Adipose-tissue-derived mesenchymal stem cells	HCC	Hepatocellular carcinoma
TwHF	Tripterygium wilfordii Hook F	siRNA	Small interfering RNA
miRNA	microRNA	mRNA	Messenger RNA
SNA	Spherical nucleic acids	DLBCL	Diffuse large B-cell lymphoma
CRCL	Chaperone-rich cell lysate	MPS	Mononuclear phagocyte system
MM	Malignant melanoma	TP	Triptolide
UC	Ulcerative colitis	RA	Rheumatoid arthritis
T-regs	T-regulatory cells	APCs	Antigen-presenting cells
MWA	Microwave ablation	EVs	Extracellular vesicles
DCs	Dendritic cells	Lung-Exo	Lung-derived exosomes
RFP	Red fluorescent protein	HEK-Exo	HEK293T-derived exosomes
hAdMSCs	Human adipose-derived mesenchymal stem cells	VEGF-A	Human vascular endothelial growth factor A
BMP-2	Human bone morphogenetic protein 2	TAgs	Toxoplasma gondii antigens
